# Septal Artery Injury During Left Bundle Branch Area Pacing: Electrophysiological Warning and Coil Embolization

**DOI:** 10.1016/j.jaccas.2026.109442

**Published:** 2026-07-28

**Authors:** Jing Song, Xueqiang Guan, Jia Li, Sheng Ye, Xuliang Chen, Jiafeng Lin, Jin Li

**Affiliations:** Department of Cardiology, Second Affiliated Hospital of Wenzhou Medical University, Wenzhou, China

**Keywords:** cardiac pacemaker, coronary angiography, electrophysiology

## Abstract

**Background:**

Left bundle branch area pacing (LBBAP) requires deep septal lead implantation and carries a risk of septal vascular injury.

**Case Summary:**

A 58-year-old man with binodal disease underwent dual-chamber pacemaker implantation using LBBAP. Initial high septal lead placement was associated with markedly elevated impedance and suboptimal pacing parameters. After repositioning, parameters normalized. However, the patient developed acute chest pain, ST-segment elevation, and ventricular tachycardia. Coronary angiography demonstrated septal branch perforation with contrast extravasation, and echocardiography confirmed an interventricular septal hematoma. Transcatheter coil embolization achieved complete hemostasis with favorable recovery.

**Discussion:**

Abnormal pacing parameters and ventricular arrhythmias may represent early electrophysiological markers of septal vascular injury during LBBAP, providing important insights into its recognition, management, and procedural implications.

**Take-Home Messages:**

Septal branch injury is a rare but potentially serious complication of LBBAP. Early recognition and prompt imaging-guided intervention are essential to ensure procedural safety.

## Visual Summary

**Figure:**
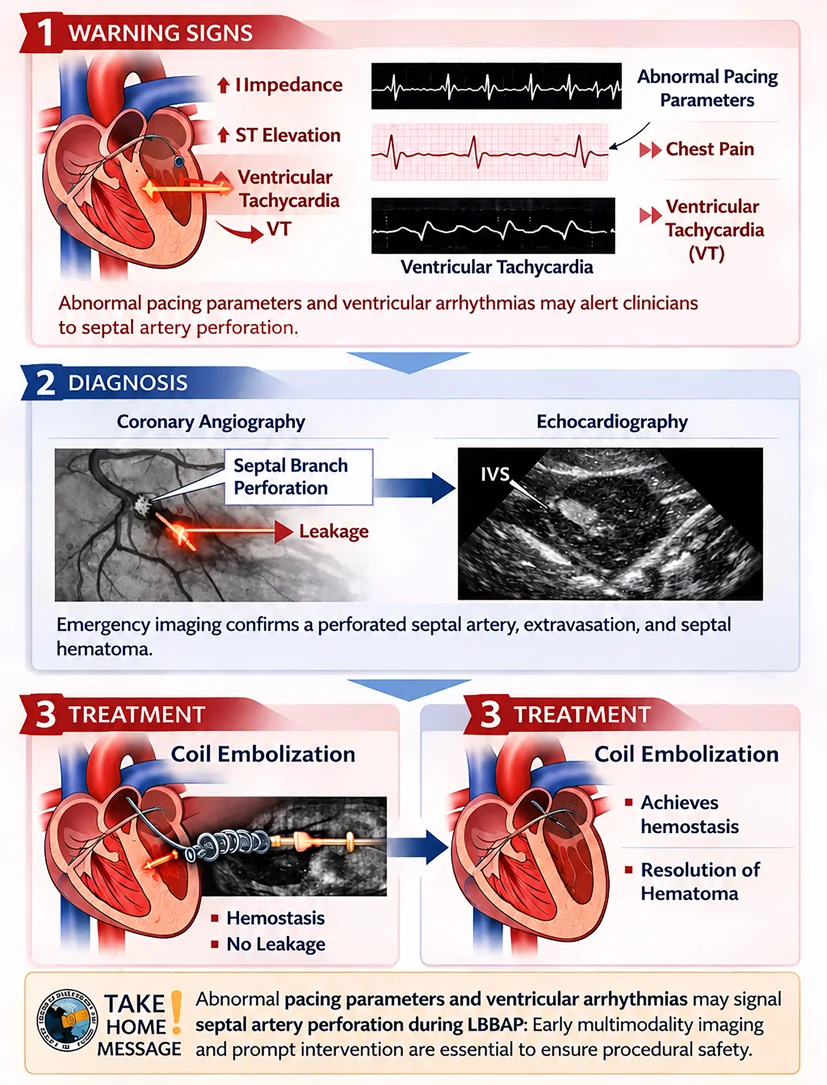

Left bundle branch area pacing (LBBAP) is increasingly used as a physiological pacing strategy that preserves ventricular synchrony.^1^ Despite its favorable electrical performance, deep septal lead implantation introduces potential risks related to adjacent septal structures, including coronary perforator arteries.^2^ Clinically significant septal vascular injury remains rare and incompletely characterized. We report a case of septal artery injury during LBBAP and highlight its early electrophysiological warning signals and management.

**Take-Home Messages**

• Septal branch injury is a rare but potentially serious complication of left bundle branch area pacing.
• Early recognition and prompt imaging-guided intervention are essential to ensure procedural safety.

## History of Presentation

A 58-year-old man presented with a 2-month history of chest discomfort and fatigue. Electrocardiogram (ECG) and ambulatory monitoring revealed sinus arrest, junctional escape rhythm, and intermittent high-grade atrioventricular block, consistent with binodal disease (Figure 1). Baseline coronary angiography revealed moderate multivessel coronary artery disease without hemodynamically significant stenosis.

**Figure 1:**
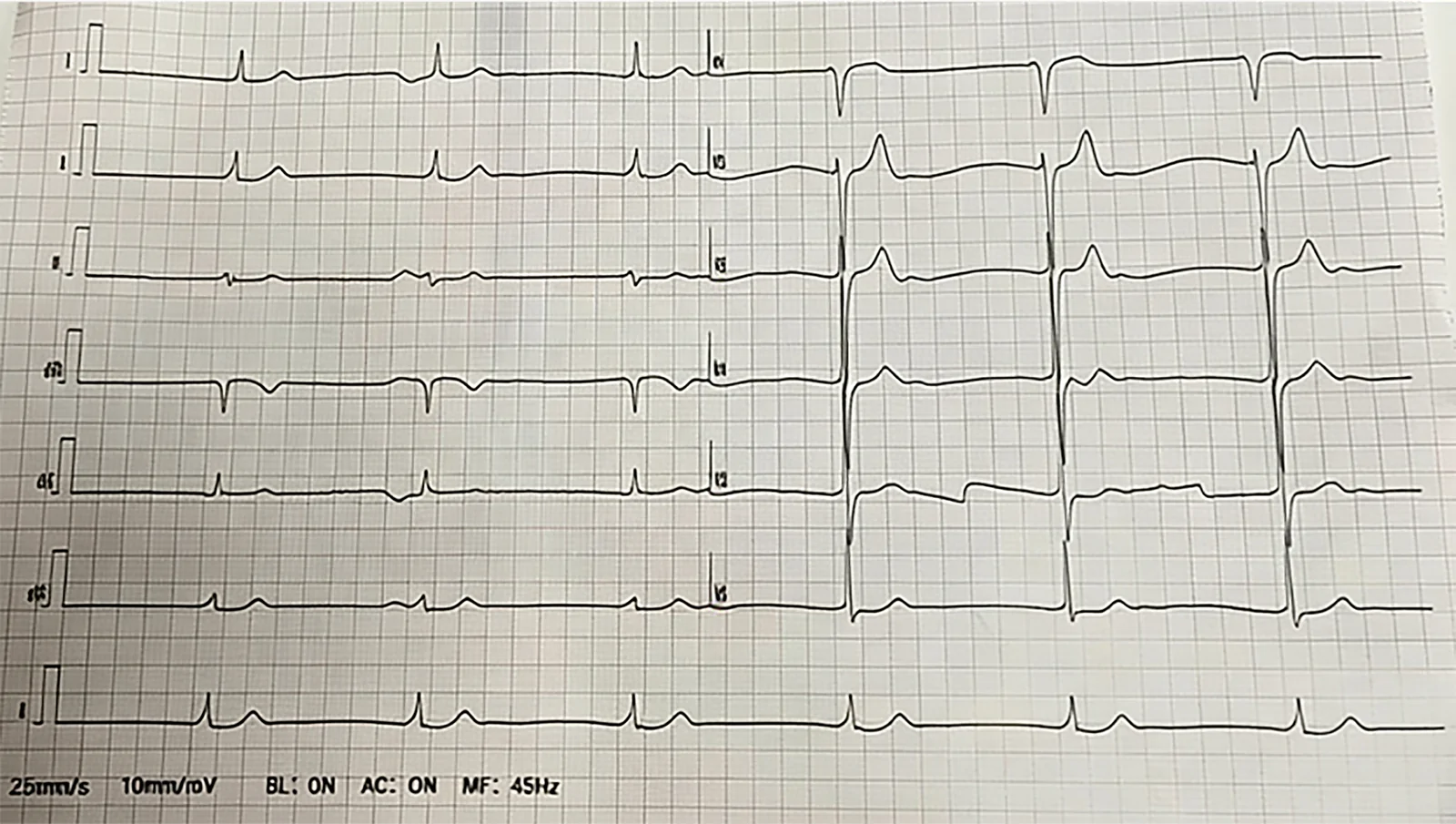
Baseline Electrocardiogram Demonstrating Sinus Arrest With Junctional Escape Rhythm

## Past Medical History

The patient's medical history was significant for type 2 diabetes mellitus and coronary artery disease.

## Differential Diagnosis

In addition to chest discomfort and fatigue due to binodal disease, other potential etiologies for presentation include acute coronary syndrome, heart failure, and noncardiac reasons for the symptoms.

## Investigations

Venous access was obtained via fluoroscopically guided puncture of the left axillary vein. An LBBAP lead (Medtronic SelectSecure 3830) was delivered using a C315HIS sheath (Medtronic). Initial mid to high septal implantation was associated with markedly elevated impedance (approximately 2,300 Ω) and a high capture threshold (4.5 V), suggesting suboptimal lead positioning. The lead was subsequently repositioned to a lower septal site, achieving satisfactory parameters, including a paced right bundle branch block morphology, sensing of 7.6 mV, capture threshold of 1.2 V, and impedance of 980 Ω (Figure 2).

**Figure 2:**
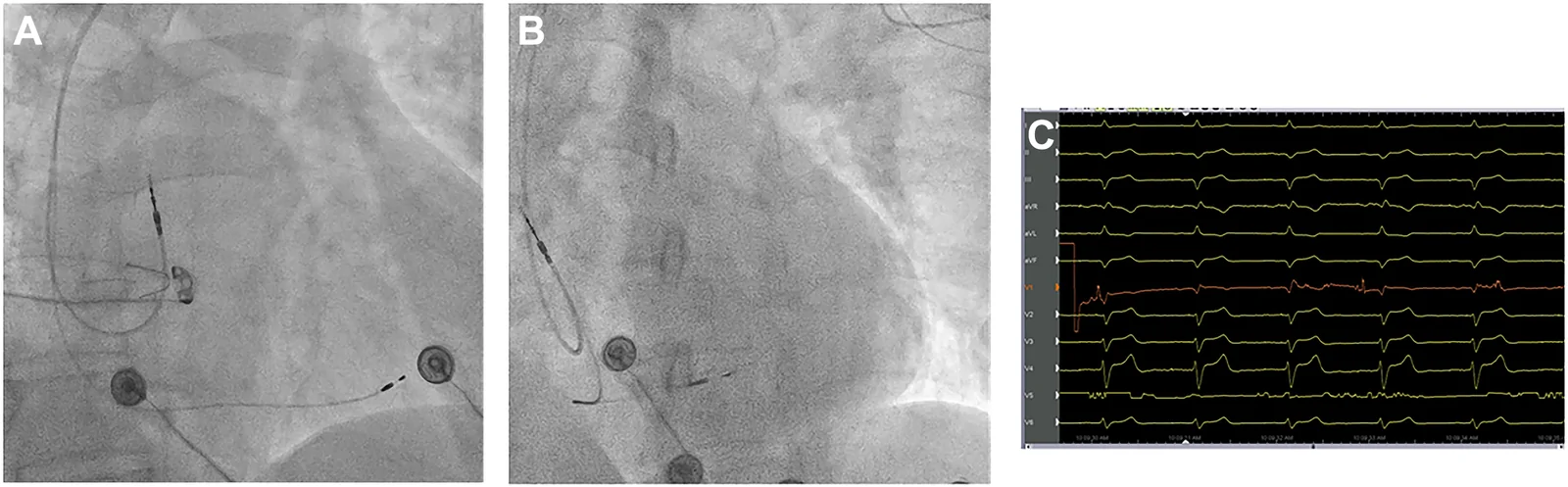
Final Lead Position and Pacing Morphology During Left Bundle Branch Area Pacing Fluoroscopic images demonstrating the final position of the left bundle branch area pacing lead in the (A) right anterior oblique projection and (B) left anterior oblique projection; (C) shows the paced electrocardiogram exhibiting a right bundle branch block morphology, consistent with effective left bundle branch area capture.

At the final stage of the procedure, the patient developed acute chest pain with diaphoresis. ECG demonstrated ST-segment elevation in leads V_1_-V_3_ and episodes of ventricular tachycardia (Figure 3). Bedside transthoracic echocardiography revealed an interventricular septal hematoma measuring approximately 10 × 6 mm without pericardial effusion (Figure 4).

**Figure 3:**
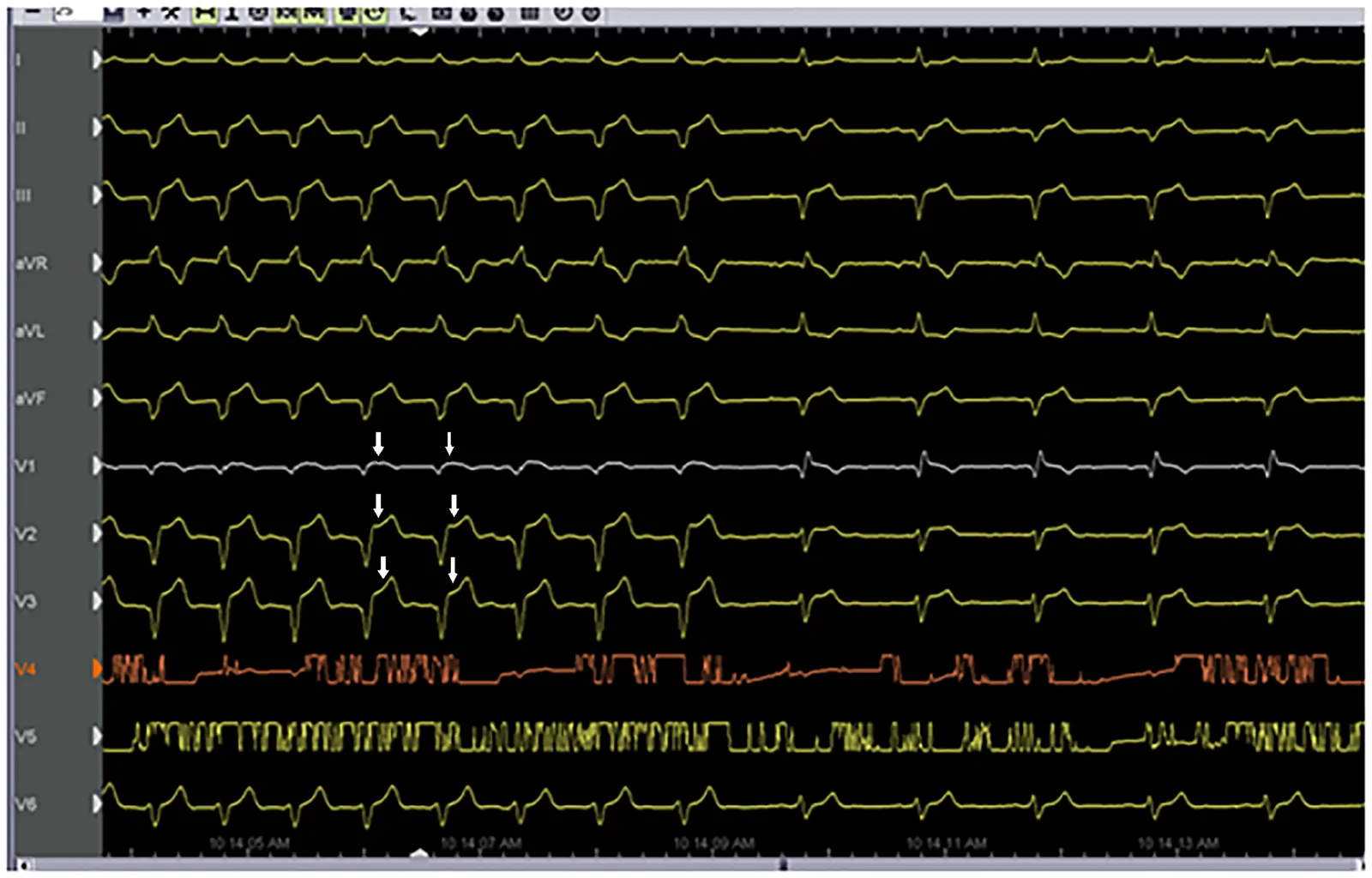
Intraprocedural Electrocardiographic Changes Showing ST-Segment Elevation and Ventricular Tachycardia Electrocardiogram recorded during the procedure demonstrating ST-segment elevation in leads V_1_-V_3_ (arrows) and ventricular tachycardia characterized by a wide-complex tachycardia pattern.

**Figure 4:**
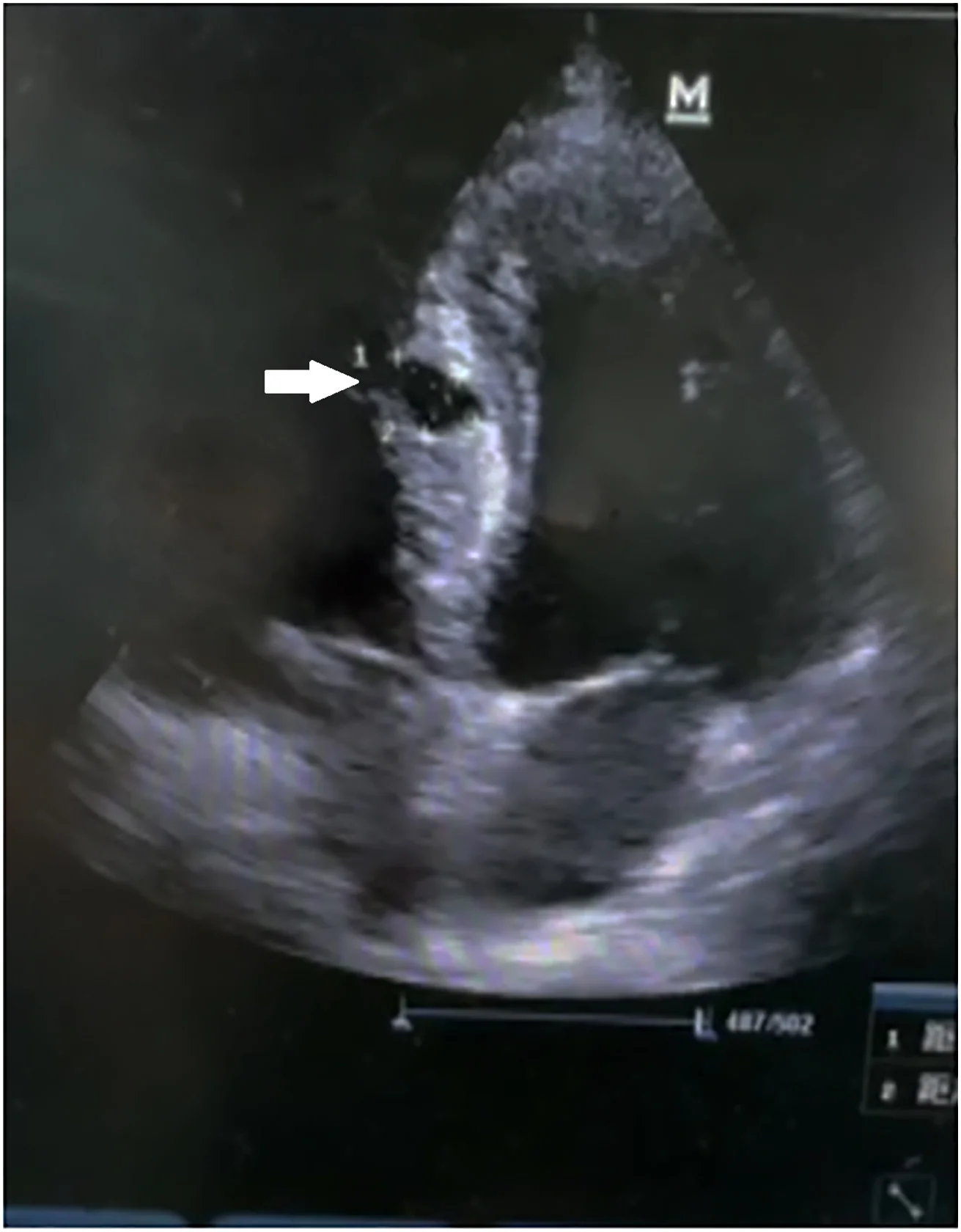
Transthoracic Echocardiography Demonstrating Interventricular Septal Hematoma Transthoracic echocardiography demonstrating a localized interventricular septal hematoma (arrow) measuring approximately 10 × 6 mm, appearing as a hypoechoic intramyocardial lesion without pericardial effusion.

## Management

Emergency coronary angiography demonstrated contrast extravasation from the third septal branch of the left anterior descending artery at the site of the initial lead deployment, consistent with septal branch perforation (Video 1, Figure 5). A microcatheter was advanced into the injured vessel, and coil embolization was successfully performed (Video 2, Figure 6). Repeat angiography confirmed complete cessation of contrast leakage.

**Figure 5:**
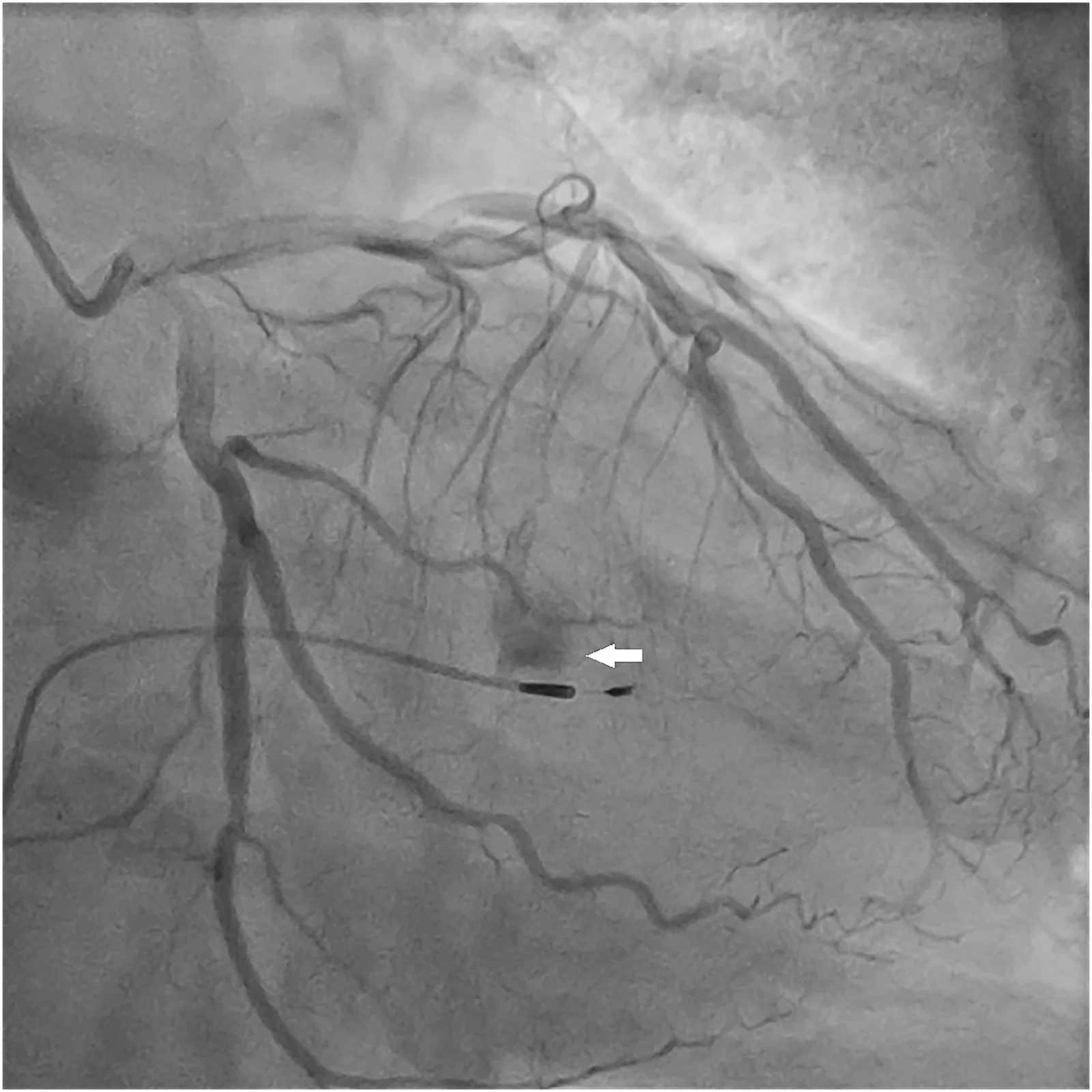
Coronary Angiography Demonstrating Septal Branch Perforation Emergency coronary angiography demonstrating contrast extravasation (arrow) from the third septal branch of the left anterior descending artery, consistent with septal branch perforation.

**Figure 6:**
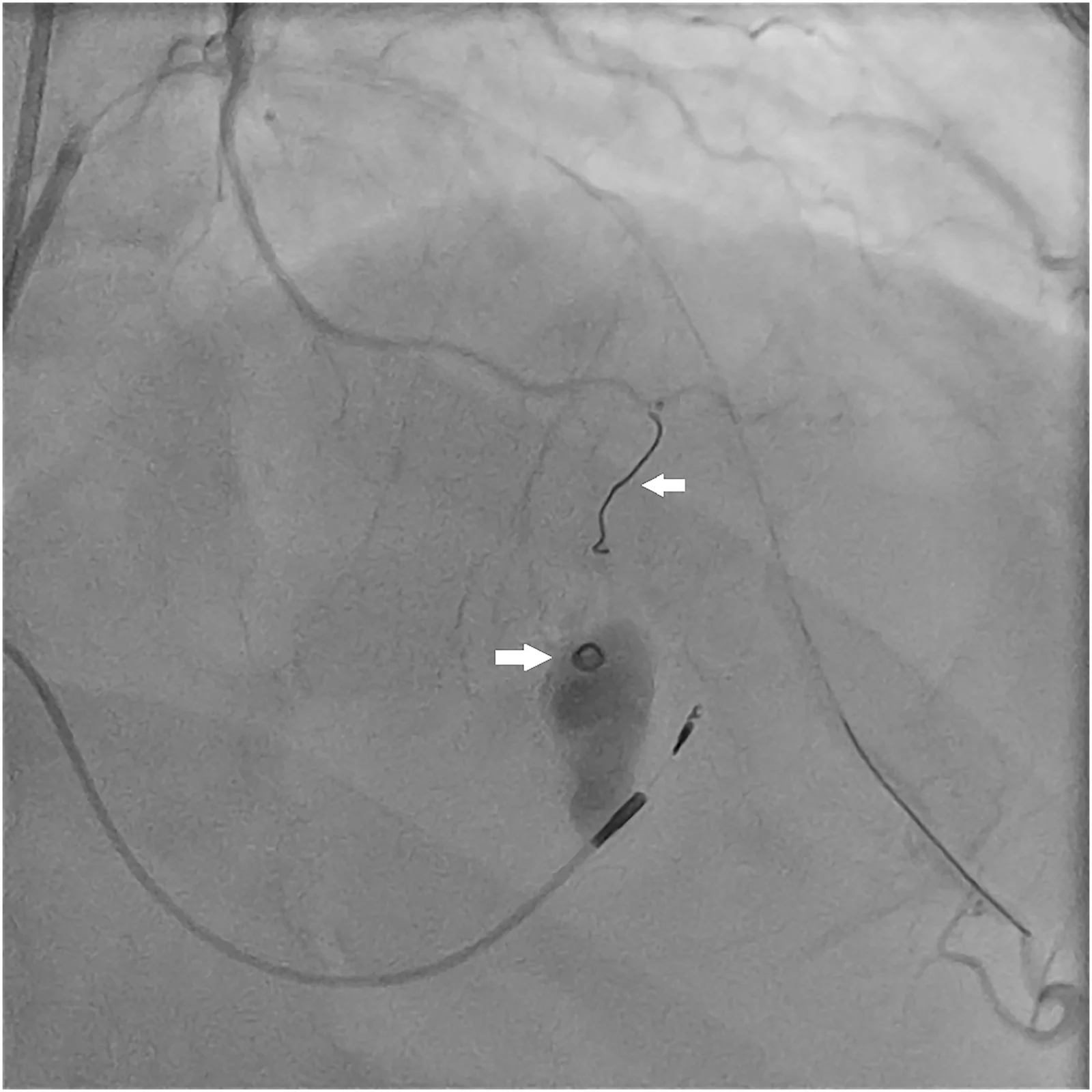
Coil Embolization of the Injured Septal Branch Coronary angiography demonstrating advancement of a microcatheter into the injured septal branch and successful coil embolization (arrow), resulting in occlusion of the perforated vessel.

## Outcome and Follow-Up

The patient remained hemodynamically stable, and follow-up imaging showed no hematoma progression. At the 1-month follow-up, the patient was asymptomatic with stable pacing parameters and no device-related complications (Figure 7).

**Figure 7:**
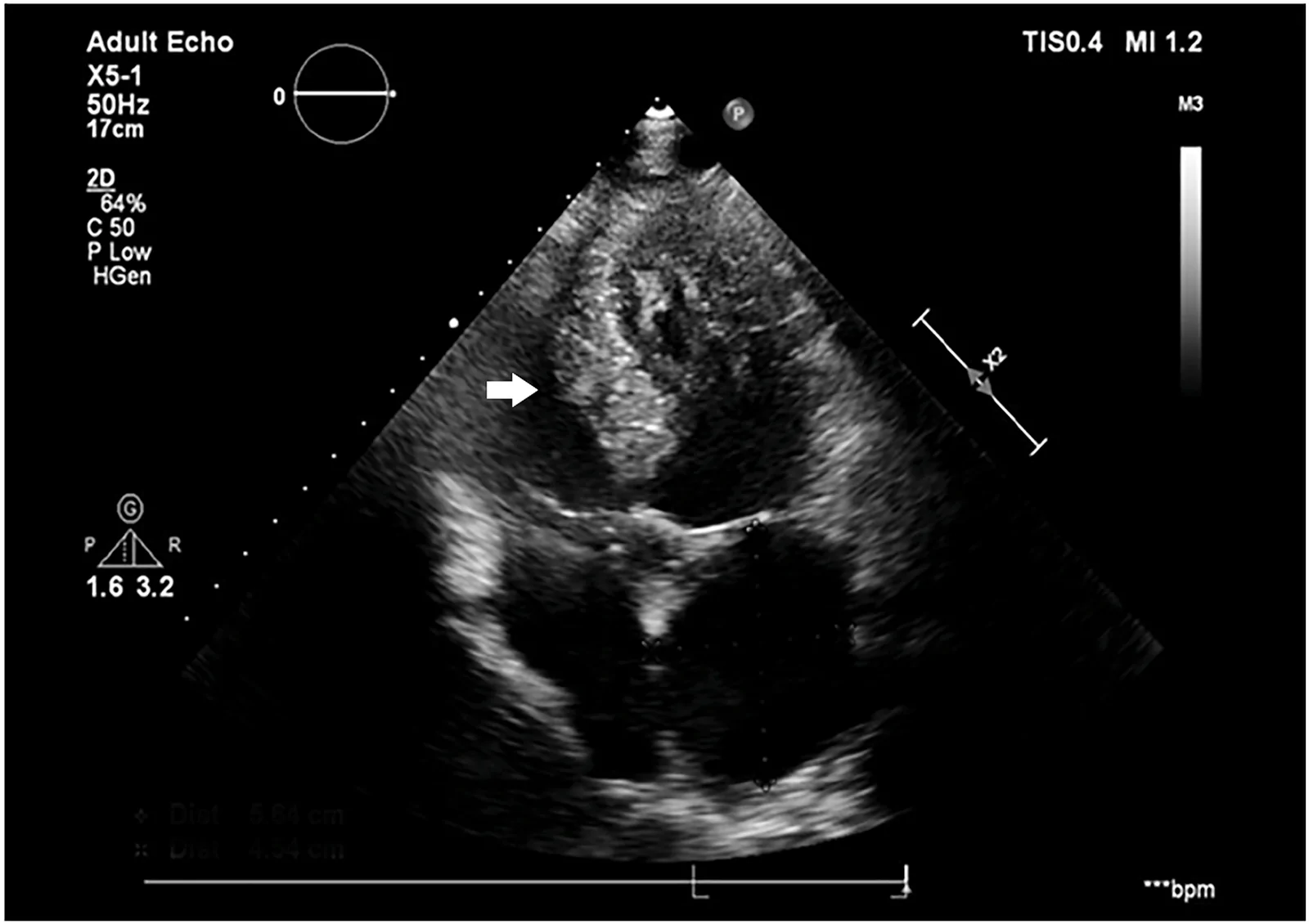
Follow-Up Transthoracic Echocardiography at 1 Month Transthoracic echocardiography at the 1-month follow-up demonstrating resolution of the interventricular septal hematoma (arrow) with preserved septal integrity and no pericardial effusion.

## Discussion

Rather than reiterating the established role of LBBAP, the present case highlights a less well-defined aspect of this technique—procedure-related intraseptal vascular injury. Although LBBAP has demonstrated favorable safety and efficacy in large cohorts, including the MELOS study, coronary septal branch injury remains insufficiently characterized and likely under-recognized in clinical practice.^3^ This gap underscores the importance of identifying early and clinically actionable warning signals.

This case illustrates septal coronary artery injury leading to interventricular septal hematoma and extends prior reports by emphasizing abnormal pacing parameters and ventricular arrhythmias as early indicators of vascular injury.^4^ Previous cases have primarily focused on late manifestations such as pericardial effusion, hemodynamic compromise, or tamponade.^5^ In contrast, our findings suggest that electrophysiological changes may precede overt structural complications, offering a critical window for early detection.

From an anatomical perspective, septal perforator arteries—typically originating from the left anterior descending artery—run in close proximity to the intended pacing target. Variability in their distribution, caliber, and trajectory results in an unpredictable “safe zone” during lead implantation. This is particularly relevant when the lead is positioned too anteriorly or at a higher septal level, increasing the likelihood of vascular injury.^6^

From a procedural standpoint, markedly abnormal pacing parameters should not be interpreted solely as technical failure. In this case, the elevated impedance and high capture threshold were observed during the initial high septal implantation and prompted immediate lead repositioning. These findings may reflect engagement of nonmyocardial structures, including vascular channels, or early tissue disruption. Importantly, these abnormalities preceded clinical deterioration and angiographic confirmation of septal branch perforation, despite normalization of pacing parameters after repositioning. Although this observation is hypothesis generating, it provides a practical electrophysiological clue that may facilitate earlier recognition of vascular injury during LBBAP.

Clinically, the combination of chest pain, ST-segment elevation, and ventricular arrhythmias during implantation should prompt immediate evaluation for coronary injury. Ventricular tachycardia in this context may result from local ischemia, intramyocardial hematoma formation, or mechanical irritation of the conduction system. Coronary angiography remains the gold standard for diagnosis, while echocardiography plays a complementary role in identifying interventricular septal hematoma and excluding pericardial complications.

Interventricular septal hematoma is an uncommon but potentially serious complication that has been reported in various clinical settings, including coronary interventions, septal ablation, and cardiac trauma. Its pathogenesis is most commonly attributed to injury of septal perforator arteries, resulting in intramyocardial bleeding.^7^ Although rare, septal hematoma may lead to outflow tract obstruction, conduction disturbances, or cardiac tamponade, necessitating close monitoring and timely intervention.^8^

Management strategies for septal vascular injury during LBBAP are not standardized. Conservative management may be appropriate in hemodynamically stable patients without active bleeding; however, ongoing contrast extravasation warrants prompt intervention. In the present case, coil embolization achieved rapid and definitive hemostasis while avoiding surgical repair, consistent with previous reports supporting its safety and efficacy.^9^ Clinical decision-making should be individualized based on clinical presentation, hemodynamic status, and imaging findings.

Finally, this case underscores the importance of integrating electrophysiological parameters with anatomical and imaging guidance to improve procedural safety. Electrocardiographic morphology, fluoroscopic positioning, and, when necessary, contrast injection should be used to optimize lead placement and avoid high-risk trajectories. Taken together, these findings support a pragmatic safety framework for LBBAP: Recognize abnormal pacing parameters as potential warning signals, maintain vigilance for intraprocedural symptoms, and initiate early multimodality evaluation to enable timely intervention.

## Conclusions

Septal vascular injury during LBBAP may be heralded by abnormal pacing parameters and ventricular arrhythmias. Early recognition and timely intervention are critical to preventing serious complications.

**Visual Summary:**
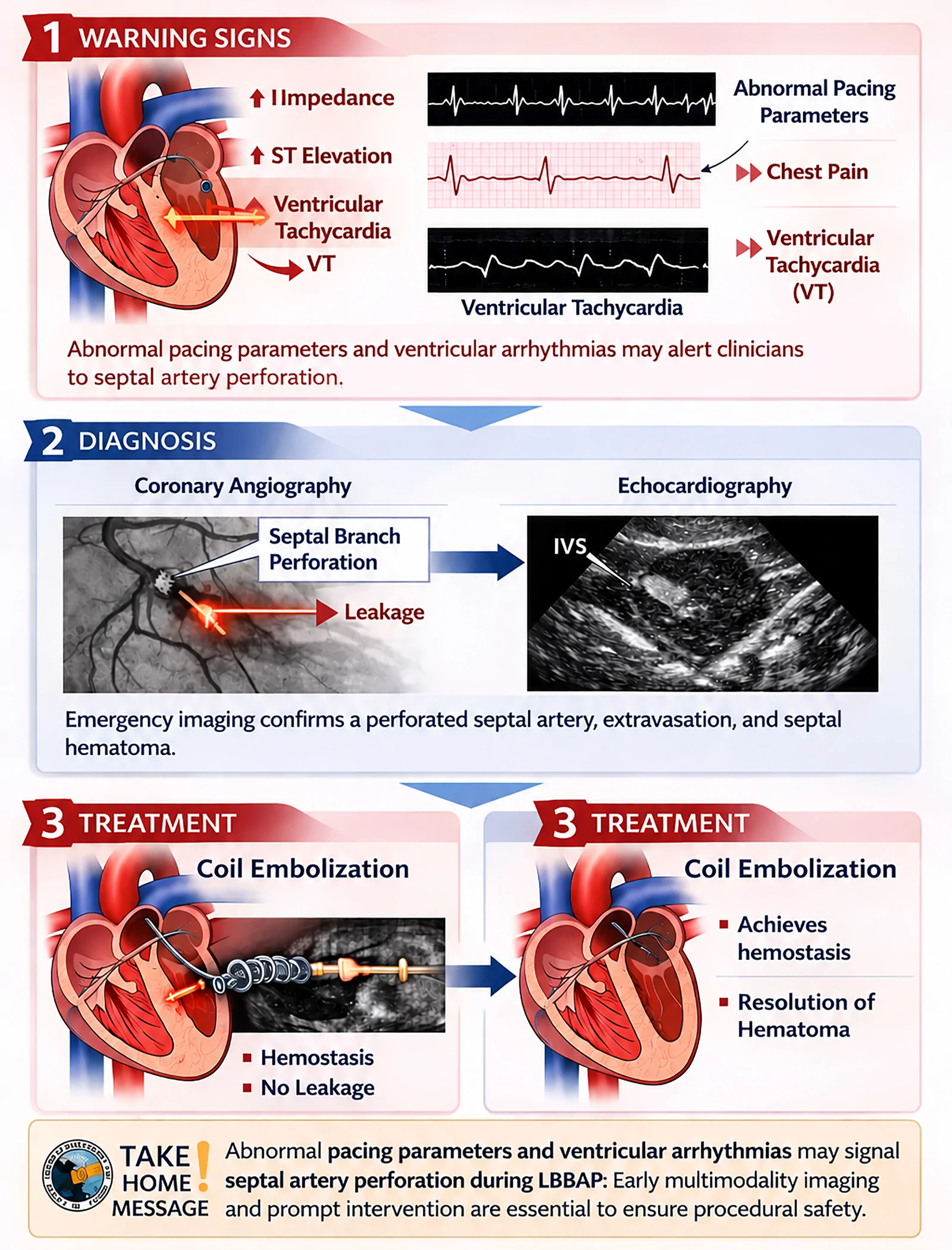
Mechanism and Management of Septal Artery Injury During Left Bundle Branch Area Pacing IVS = interventricular septum; LBBAP = left bundle branch area pacing; VT = ventricular tachycardia.

## Funding Support and Author Disclosures

The authors have reported that they have no relationships relevant to the contents of this paper to disclose.

## Appendix

Video 1 Coronary Angiography Demonstrating Contrast Extravasation From a Septal Branch of the Left Anterior Descending Artery

Video 2 Selective Microcatheter Positioning and Coil Embolization of the Injured Septal Branch

## References

[1] Glikson M., Burri H., Abdin A. (2025). European Society of Cardiology (ESC) clinical consensus statement on indications for conduction system pacing, with special contribution of the European Heart Rhythm Association of the ESC and endorsed by the Asia Pacific Heart Rhythm Society, the Canadian Heart Rhythm Society, the Heart Rhythm Society, and the Latin American Heart Rhythm Society. Europace.

[2] Raza M.H., Maqbool M.U., Kumar S., Malik J., Ali S., Khan Z.A. (2023). Coronary artery complications with left bundle branch area pacing: a review of literature. Pacing Clin Electrophysiol.

[3] Jastrzebski M., Kielbasa G., Cano O. (2022). Left bundle branch area pacing outcomes: the multicentre European MELOS study. Eur Heart J.

[4] Konig S., Hilbert S., Sulimov D., Kister T., Marinov K., Bode K. (2025). Need for temporary mechanical circulatory support for dry tamponade caused by interventricular septal hematoma: a case report of a rare complication following left bundle branch area pacing. Heartrhythm Case Rep.

[5] Chen X., Lu H., Xu L. (2023). Interventricular septal hematoma with pericardial effusion after left bundle branch pacing implantation. JACC Clin Electrophysiol.

[6] Ghosh A., Sekar A., Sriram C.S., Sivakumar K., Upadhyay G.A., Pandurangi U.M. (2024). Septal venous channel perforation during left bundle branch area pacing: a prospective study. Europace.

[7] Vargas-Barron J., Roldan F.J., Romero-Cardenas A. (2009). Dissecting intramyocardial hematoma: clinical presentation, pathophysiology, outcomes and delineation by echocardiography. Echocardiography.

[8] Zheng R., Wu S., Wang S., Su L., Ellenbogen K.A., Huang W. (2021). Case report: interventricular septal hematoma complicating left bundle branch pacing lead implantation. Front Cardiovasc Med.

[9] Baszko A., Zabicki B., Danowski R., Telec W., Malek-Elikowska M. (2024). Ventricular septal hematoma caused by left bundle branch pacing electrode implantation. The role of coils in closing abnormal vascular connections. Kardiol Pol.

